# Inhibitory Dimensions and Delay of Gratification: A Comparative Study on Individuals with Down Syndrome and Typically Developing Children

**DOI:** 10.3390/brainsci11050636

**Published:** 2021-05-14

**Authors:** Martina Fontana, Maria Carmen Usai, Sandra Pellizzoni, Maria Chiara Passolunghi

**Affiliations:** 1 Department of Life Sciences, University of Trieste, 34127 Trieste, Italy; martina.fontana@phd.units.it (M.F.); passolu@units.it (M.C.P.); 2 Department of Education Sciences, University of Genoa, 16126 Genova, Italy; maria.carmen.usai@unige.it

**Keywords:** Down syndrome, response inhibition, interference suppression, delay of gratification, hot and cool executive functions, chronological age

## Abstract

While previous research on inhibition in people with Down syndrome (DS) reported contradictory results, with no explicit theoretical model, on the other hand, a more homogeneous impaired profile on the delay of gratification skills emerged. The main goal of the present study was to investigate response inhibition, interference suppression, and delay of gratification in 51 individuals with DS matched for a measure of mental age (MA) with 71 typically developing (TD) children. Moreover, we cross-sectionally explored the strengths and weaknesses of these components in children and adolescents vs. adults with DS with the same MA. A battery of laboratory tasks tapping on inhibitory sub-components and delay of gratification was administrated. Results indicated that individuals with DS showed an overall worse performance compared to TD children on response inhibition and delay of gratification, while no differences emerged between the two samples on the interference suppression. Additionally, our results suggested that older individuals with DS outperformed the younger ones both in response inhibition and in the delay of gratification, whereas the interference suppression still remains impaired in adulthood. This study highlights the importance of evaluating inhibitory sub-components considering both MA and chronological age in order to promote more effective and evidence-based training for this population.

## 1. Introduction

Down syndrome (DS) is the most common neurogenetic syndrome associated with intellectual disability that affects over 1:700 live births [1,2]. Given the progressive increase in the life expectancy of people with DS, currently reaching nearly 60 years [3], further research is necessary to better understand the neuropsychological developmental trajectories of individuals with DS, considering both their mental age (MA) and their chronological age (CA). Previous studies reported that people with DS show some relatively preserved abilities with respect to visual processing, emotion recognition, and social behavior [4,5]. On the other hand, relative weaknesses are confirmed in some aspects such as verbal processing, self-regulation, executive functions (EFs), fine and gross motor functioning, motor planning, and adaptive behavior with different degrees of impairment [4,6,7,8]. While there is a widespread agreement on more pronounced difficulties on EFs as a whole [4,7], contradictory findings emerged when inhibitory skills are investigated. Therefore, the main goal of our research is to analyze inhibitory abilities in individuals with DS in greater depth by elucidating patterns of strengths and weaknesses across the lifespan.

EFs are a complex set of cognitive abilities necessary for emotion regulation, deliberate reasoning, and self-regulation that are required to reach suitable levels of adaptive functioning, quality of life, and wealth, often more than IQ scores and socioeconomic status [9,10,11,12]. In fact, the presence of difficulties in EFs can be considered as a transdiagnostic indicator of atypical development [13]. The literature agrees on the fact that EFs can be distinguished into three core components: updating, inhibition, and shifting [14], which support higher-order cognitive processes such as problem-solving or planning [14,15]. The developmental trajectories of EFs consist of relative plasticity of these functions over an extended range of time (i.e., from infancy to early adulthood) with greater levels of plasticity from early childhood to adolescence [16].

There is considerable behavioral and neural evidence that EFs vary along a continuum from hot to cool, which typically work together to solve real-world problems [17,18]. While hot EFs are elicited in motivational and emotional contexts, cool EFs are required in cognitively demanding situations. Some studies suggested that the distinction between hot and cool EFs can be observed in TD children’s behavior by the age of 3–4 [19,20], while others found these two factors as separate in children starting from the age of 24 months [21,22]. The evidence clearly highlights the importance to jointly analyze both hot and cool EFs given their importance for some crucial aspects such as daily life, emotion regulation, and academic outcomes both in children [20,23,24] and adolescents [19,25].

### 1.1. Developmental Trajectories of Inhibition and Delay of Gratification in Typical Development

Inhibition, one of the three core components of EFs, refers to the ability to control one’s mental processes and responses, ignoring interfering stimuli, and to perform an alternative action [10]. It is commonly described as a multi-componential construct that includes several types of functions [26]. Evidence suggests that a two-factor model composed of response inhibition (i.e., the ability to suppress automatic behavioral responses in order to provide the correct answer) and interference suppression (i.e., the ability to ignore distracting information or to suppress competing response tendencies) best describes inhibition in children of 4 years of age [27], 5 to 6 years old [28], and in younger and older adults [29]. Interference suppression may emerge after response inhibition and is considered as a more complex dimension that requires greater involvement of working memory (WM) [27,30]. In fact, while response inhibition could be considered as a simpler form of inhibition that is usually assessed with paradigms such as Go/no-go, Day-Night Stroop, Circle Drawing task, and delay of gratification, the more complex interference suppression is significantly predicted in tasks such as the Flanker and the Hearts and Flowers [27,31].

On the other hand, the delay of gratification that assess the simpler form of inhibition is considered as a hot dimension [31,32,33] in which individuals have to resolve the conflict to avoid a more salient and immediate reward (i.e., smaller reward) to approach a less salient delayed reward (i.e., larger but delayed reward). Delay of gratification skills is usually assessed with tasks such as the Marshmallow task [34] or the Wrap Delay task, or the Gift Delay task [35]. Literature on the developmental delay of gratification trajectories suggested that better performance could be detected by four years of age, coinciding with the development of some cool EF skills such as the ability to resolve the conflict and to shift between attention sets [17]. Likewise, to reach better waiting time on the delay of gratification tasks, subjects have to learn to reduce the salience of the immediate reward using specific strategies such as self-talking and distraction [24]. Literature on typically developing (TD) children reports that those who are less capable of delaying gratifications showed higher levels of behavioral problems such as hyperactivity, impulsivity, and conduct problems [25]. Furthermore, given the longitudinal stability of the delay of gratification pattern, it seems to be a predictor of academic, cognitive, and social future outcomes [36,37].

### 1.2. Inhibitory Sub-Components and Delay of Gratification in People with Down Syndrome

Inhibitory skills in people with DS play a fundamental role in a multitude of crucial outcomes such as daily life autonomy, adaptive and social behavior, academic achievements, and occupational perspectives [8,38,39]. In the last decades, an increasing amount of research is focusing on EFs, indicating overall poorer performance for people with DS compared to the TD group [40], whereas studies on the inhibitory construct in people with DS have reported contradictory findings. Some cross-sectional studies that examined inhibition using informant-report measure, such as the Behavior Rating Index of Executive Function (BRIEF) [41], suggested that in individuals with DS aged 4-to-24’s inhibition may improve with age [42], whereas others indicated that inhibitory abilities were consistent from 2 to 18 years of age and then declined around age 30 [43].

Moreover, while some studies have identified inhibitory difficulties in the sample with DS when they were rated by their carers, but not by their teachers (e.g., [44,45]), other research reported these difficulties only when teachers were the raters instead of carers (e.g., [39]), while even other authors reported that both parents and caregivers assessed inhibition as relatively impaired compared to other EFs (e.g., [46]). As reported by Gioia et al. [41], only small to medium correlations emerged between parents’ and teachers’ reports, suggesting that performance on EFs may be interpreted differently by the two raters and that EF demand may vary across the school and home environments.

In a similar way, comparing literature that assessed inhibition in individuals with DS using laboratory tasks, the same contradictory trend appeared. In fact, while in some studies people with DS showed worse performance on inhibitory tasks compared to the TD control group (e.g., [7,47,48]), in other research, no differences emerged between the group with DS and the TD control group (e.g., [49,50]), while in other studies that used more than one task to assess different inhibitory dimensions mixed results were recorded (e.g., [4,28,51]). Moreover, it should be considered that whereas TD children’s inhibitory sub-components have been analyzed and assessed referring to a theoretical model (see [27]), the same cannot be said for studies on inhibition in people with DS. Recently, trying to fill this gap, Fontana and colleagues [52] conducted a meta-analysis on inhibition abilities in people with DS, indicating on average moderately impaired inhibitory performance in this population when they were matched for MA with TD children, whereas when the two groups were not matched for MA, they did not significantly differ from each other. These results highlight the importance of considering the following factors in assessing inhibition: proxies for MA, different inhibitory tasks to assess the diverse inhibitory sub-components (e.g., response inhibition, interference suppression or proactive interference, and delay of gratification), and smaller ranges of CA.

Nevertheless, literature that analyzed the simpler form of inhibition (e.g., response inhibition or prepotent response inhibition) demonstrates high levels of variability. For example, Daunhauer et al. [4] and Will et al. [8], using the “Simon says” paradigm, showed significant differences in inhibition performance between the group with DS and the TD control group, while Costanzo et al. [53] and Traverso et al. [28], assessing inhibition with the Go/no-go task, reported no significant differences between the two groups in accuracy scores. Otherwise, differences also emerged using the same inhibitory task. For example, considering accuracy score, while some studies reported significant differences in performing the Stroop-like task (e.g., [7,51]), other studies reported comparable performance between the two groups (e.g., [53]). Substantial differences with opposite results also emerged in performing the Stroop-like task when response time (RT) was considered ([51,53]).

Regarding tasks tapping on more complex components, Borella et al. [51], using the Proactive Interference task and the Direct Forgetting task, reported significant more intrusions in the group with DS, although Traverso et al. [28], administrating the Fish Flanker and the Dots tasks, found no differences in interference suppression accuracy score between the group with DS and the TD control group, instead, longer RTs were recorded for the sample with DS but only for the Flanker task.

Finally, there is a widespread agreement on difficulties for people with DS on delaying gratification, reporting overall a shorter waiting time. While Daunhauer et al. [4] identified no significant differences between children with DS and the TD control group in the Snack Delay task, Daunhauer et al. [54], using the same task, suggested greater difficulties for people with DS. Moreover, Daunhauer and colleagues [54] identified a significant negative correlation between the scarce performance on the Delay of Gratification task and academic performance such as letter-word identification and applied problems with potentially far-reaching implications for future academic achievement for students with DS. Other studies that focused on the delay of gratification in children and young adults with DS found that the first group had significantly shorter waiting times and that 45% of the sample waited less than three minutes to receive their reward [55,56]. Furthermore, Cuskelly et al. [57] registered worse performance on delay of gratification tasks in young adolescents with DS matched for MA to TD children, suggesting an association between the delay of gratification deficit and receptive language.

### 1.3. The Present Study

The previous paragraph described the state-of-art literature on EFs in typical development and in individuals with DS, showing that studies that assessed inhibitory performance in people with DS: (a) are quite inconsistent; (b) assessed this specific component with a limited number of tasks and without referring to a theoretical model (excepted for [28,51]); (c) included all measures under the more general label of “inhibition”, (d) focused more on children and adolescent with DS, leaving these components in adults with DS less investigated still.

To fill these gaps in literature, this study aims to:

(1) Investigate response inhibition, interference suppression [27,28,29], and delay of gratification in people with DS compared to TD children matched for a measure of MA using a specific battery that assess different inhibitory sub-components [28,58];
(2) Try to add some information on cross-sectional developmental trajectories of inhibitory sub-components and delay of gratification in a sample of people with DS clustered in two different groups on the basis of their CA.

Concerning the comparison between the sample with DS and TD children matched for MA, we expected to find worse performance in those with DS in response inhibition, interference suppression, and delay of gratification tasks [28,57]. Considering instead only the group of individuals with DS, we expected worse performance on average in children and adolescents with DS (DS1) compared to adults with DS (DS2), in particular on interference suppression, which is the more complex dimension (according to [43]).

## 2. Materials and Methods

### 2.1. Participants

A final sample of 122 individuals took part in this research. The study enrolled 51 individuals with DS (25 females and 26 males) with a mean CA of 23.73 years (*SD* = 12.24, age range = 5.5–54.5 years) and a mean MA of 6.73 years (*SD* = 1.73), and 71 TD children (39 females and 32 males) with a mean CA of 6.17 years (*SD* = 0.72, range = 4.9–8.0 years) and a mean MA of 7.13 (*SD* = 1.23). We excluded from our sample individuals with DS with: (a) trisomy 21 with mosaicism; (b) a comorbid diagnosis of Autism Spectrum Disorder; (c) severe visual or hearing impairments; (d) neurological impairments or developmental disabilities or a combination of these.

The Colored Progressive Matrices Test (CPM, [59]) was administrated as a screening measure to match the group with DS with the TD control group for nonverbal reasoning ability. A one-way ANOVA on participants’ raw CPM scores indicated no significant differences (*F =* 2.30, *p =* 0.13) between the group with DS and the TD control group.

To deeply investigate the performances of the group with DS, we split this group by CA considering the CA of 18 years old as cut-off, according to the literature on EFs in TD that show significant changes and improvement until late adolescence [18] and developmental stability on EFs for people with DS from 2 to 18 years old [43]. Therefore, the sample with DS consisted of the following two groups: 21 children and adolescents with DS (DS1) with a mean CA of 12.27 years (*SD* = 3.71), and 30 adults with DS (DS2) with a mean CA of 31.75 years (*SD* = 9.35). A one-way ANOVA showed significant differences in participants’ CA (*F =* 81.66, *p =* 0.0001) between the two groups.

### 2.2. Procedure

Each participant was individually assessed by trained psychologists in a quiet room in three separate sessions, each lasting about 30–40 min with an interval of 3–4 days. According to Carlson and Moses [60], the task order was maintained constant to better investigate and control individual differences. The group with DS was assessed in two Associations for individuals with DS in northern Italy, while the TD control group belonged to kindergartens and primary schools of northern Italy.

This study was conducted in accordance with the recommendations of the Ethical Code of the Italian Register of Professional Psychologists and of the Ethical Guidelines of the Italian Association of Psychology with written informed consent from all subjects, in accordance with the Declaration of Helsinki. In addition, the administrators at the Associations for people with DS provided their consent to take part in the research and to use their educational institutions for the administration of the test (Prot. n. 1118, April 2017). Regarding the TD control group, consent to involve in the research was obtained both from schools and parents.

### 2.3. Measures

A battery of eight tasks was presented both to the group with DS and to the TD control group to assess inhibition (i.e., response inhibition and interference suppression) and delay of gratification abilities. All measures are well-known inhibitory tasks that have been previously used both with people with DS and with TD children without showing any floor or ceiling effect in the MA included in this study [28,57,61]. Moreover, the tasks included in the battery do not require a verbal response and thus reduced confounding language-based influences [62,63].

#### 2.3.1. Response Inhibition Tasks

Circle Drawing task (CDT, [64]). The CDT assesses response inhibition and the ability to control and slow down a motor response. In this task, the participant is required to trace with her/his finger a circle depicted on a cardboard square (17 cm in diameter). The CDT provides for the following two conditions: the first (T1) with neutral instruction (i.e., “Trace the circle with your finger”) and the second (T2) with inhibitory instruction (i.e., “Trace the circle again, but this time as slowly as you can”). The score is calculated as the proportion of slowdown with the following formula: T1−T2/T1+T2. The test-retest reliability was 0.93 [58].

Preschool Matching Familiar Figure task (PMFFT, [65] adapted from [66]). This task evaluates the ability to control impulsive responses by shifting attention from the target figure to all the other figure stimuli [27]. The participant is asked to perform 14 trials, selecting which figure is identical to the target one among five alternatives. The number of errors (PMFFT errors, expected range 0–56) and RT (PMFFT time, expected range 0–no limit) was recorded. Cronbach’s alpha was 0.67 for PMFFT errors and 0.95 for PMFFT time for TD children [65], while it was 0.85 for the PMFFT errors and 0.94 for PMFFT time for people with DS [28].

Go/no-go task (GNG, adapted from [67]). This task is a well-known paradigm that assesses response inhibition both in children and in adults [68,69]. Sitting in front of a computer screen, the participant has to press the space bar according to specific instructions given by the examiner (e.g., “Press the space bar when blue figures appear, while do not press the space bar when a red figure turns up on the computer screen”). The task consists of the following three conditions: GNG1 (no-go, 6 red figures), GNG2 (no-go, 6 ball figures), GNG3 (no-go, 8 blue stars). Stimuli duration was 3.000 ms, and the blank page after each stimulus lasted 1.000 ms. The sum of the correct no-go responses (GNG1, expected range 0–6; GNG2, expected range 0–6; GNG3, expected range 0–8) and RT of the no-go items (expected range 0–no limits) was recorded for each condition. Cronbach’s alphas were 0.71 in the TD group and 0.83 in the group with DS [28].

Grass/snow task. This Stroop-like task assesses the ability to inhibit a prepotent response by having the subject perform an alternative action by pointing instead of speaking. The participant is presented with two square cardboards, one colored green as grass and one colored white like snow. In the first condition, the participant has to tap on the green square when she/he hears the word “grass” while tapping on the white square when the word “snow” is pronounced by the experimenter (expected range 0–16). In the second condition, the incongruent one, they had to do the opposite (i.e., to tap on the white square for “grass” and to tap on the green square for “snow”). In each condition, words are presented by the examiner in pseudo-random order. The incongruent condition was considered for the scoring, specifically the number of correct answers (expected range 0–16) and RT. In previous research with TD children, this task showed suitable reliability and construct validity with a Cronbach’s alphas of 1.00 for accuracy score [60].

#### 2.3.2. Interference Suppression Tasks

Fish Flanker task. This is a well-known paradigm that assesses interference suppression [70,71]. Sitting in front of a computer screen, the participant is required to respond to a left- or right-oriented fish by pressing a left or right response button on the keyboard. Two other fish flank the central target fish and can be directed in the same (congruent condition, 16 items) or opposite direction (incongruent condition, 16 items). After four training items (two congruent and two incongruent), 32 items were randomly presented (16 for each condition, 16 right and 16 left). A warning cross (500 ms in duration) preceded each stimulus, and then the screen turned blank after the response (500 ms). Accuracy (expected range 0–16) and RT (0–no limits) in the incongruent condition were recorded. Test-retest reliability for accuracy score was 0.50 [58].

Hearts and Flowers task. This task, also labeled as the Dots task, is a high cognitive task that assesses interference suppression [72]. A heart or a flower appears on the computer screen, and the participant has to press on the keyboard the button on the same side when a heart appeared and press the button on the opposite side when they see a flower. After a brief training session, hearts and flowers appear in a randomized presentation. Accuracy (expected range, 0–20) and RT were recorded. Test-retest reliability was 0.62 (*p* < 0.001) for accuracy score and 0.72 (*p* < 0.001) for RT [58].

#### 2.3.3. Delay of Gratification Tasks

Wrap Delay task. This task measures the ability to delay gratification, inhibiting and regulating undesirable behaviors [60]. The experimenter tells the participant they have a gift for her/him but that the gift has not been wrapped yet. The participant is told that she/he should not turn until the examiner has finished. The examiner noisily wraps the gift for over 60 s and, after that, delivers the gift to the participant. The latency to the first peek (Wrap delay latency time, expected range 0–60 s) was recorded. The test-retest reliability (Pearson’s r) was 0.95 for latency time [73].

Marshmallow task. This well-known task evaluates the ability to delay gratification and to self-regulate behaviors both in TD children and in people with DS [57,74]. The subject is presented a box that contains treats, and she/he has to make their selection. After that, the experimenter places two treats on one plate and 10 treats on another plate to ensure that participants prefer the larger amount. The participant is informed that the examiner has to leave the room to do some work and is told that if she/he waits without eating any of the treats until the experimenter will be back in the room, they could receive the larger pile. If she/he could not wait, the possibility to ring a bell to get the examiner back in the room is given, but in that case, she/he will receive the smaller pile of treats. The task ends when participants ring the bell or eat the treats, or when the time of five minutes lapses and the examiner comes back in the room. Waiting time (0–5 min) was recorded. The test-retest reliability was 0.99 for latency time scores [39].

## 3. Results

A series of multivariate analyses of variance (MANOVA) were run to assess possible differences between the group with DS and the TD control group matched for MA. We included the groups as a factor and both inhibitory and delay of gratification tasks as dependent variables. Furthermore, to analyze inhibitory and delay of gratification performances in-depth, we split the group with DS into two sub-groups based on CA. For this reason, we conducted a multivariate analysis of covariance (MANCOVA), including CA as a factor, inhibitory and delay of gratification tasks as dependent variables, and the raw CPM score as a covariate. To compare performance differences between groups, η_p_^2^ was used as a measure of effect size. The criteria of Cohen [75] were used to classify the effect sizes: small effect: η_p_^2^ = 0.01; medium effect: η_p_^2^ = 0.06; and large effect: η_p_^2^ = 0.14.

Descriptive statistics and differences between the group with DS and the TD control group using MANOVA on inhibitory and delay of gratification tasks are shown in Table 1. The MANOVA results reveal a significant main effect for group factor (Wilks’ Lambda = 0.29, *F*(17, 104) = 14.78, *p* = 0.000, η_p_^2^ = 0.70) since the two groups significantly differ from each other. With respect to accuracy scores, the TD group outperformed the sample with DS on the following tasks that assess response inhibition: CDT, PMFFT, Grass/snow task, Go/no-go 2 task, Go/no-go 3 task. Moreover, the group with DS showed worse performance compared to TD children in tasks that measured delay of gratification abilities: Wrap Delay task and Marshmallow task. No significant differences in accuracy scores emerged in the Go/no-go1 task and in the two tasks tapping on interference suppression (i.e., Fish Flanker task and Hearts and Flowers task). Regarding RT, the sample with DS showed longer RT compared to TD children on: PMFFT, Go/no-go 2 task, Go/no-go 3 task, Fish Flanker task. No significant differences emerged between the two groups on RT in the following tasks: Grass/snow task, Go/no-go 1 task, and Hearts and Flowers task.

### 3.1. Investigating Inhibitory and Delay of Gratification Differences Between the Two Groups with Down Syndrome with Different Chronological Ages

As shown in Table 2, significant mean differences emerged between the group DS1 and DS2 on response inhibition and delay of gratification tasks, while no difference emerged on performances on interference suppression tasks. More specifically, the MANCOVA results reveal a significant effect of MA (Wilks’ Lambda = 0.47, *F*(17, 32) = 2.11, *p* = 0.033, η_p_^2^ = 0.53) and CA (Wilks’ Lambda = 0.48, *F*(17, 32) = 2.00, *p* = 0.044, η_p_^2^ = 0.52) between the two groups. Analyzing accuracy scores, adults with DS (DS2) significantly outperformed children and adolescents with DS (DS1) on both response inhibition and delay of gratification measures: PMFFT, Grass/snow task, Go/no-go 1 task, Go/no-go 2 task, Go/no-go 3 task, Wrap Delay task, and Marshmallow task. No significant differences in accuracy scores emerged in the CPM, indicating substantially the same nonverbal reasoning abilities between the two groups despite different CA, CDT and in interference suppression tasks (i.e., Fish Flanker task and Hearts and Flowers task).

Regarding RT, significant differences between the two groups emerged only in the Go/no-go 1 task and in the Go/no-go 3 task. No significant differences emerged on all others RT tasks.

## 4. Discussion

The present study aimed to assess inhibitory sub-components (i.e., response inhibition and interference suppression) and delay of gratification with a specific battery of eight tasks in a sample of 51 individuals with DS matched with 71 TD children for MA. Moreover, to the best of our knowledge, these results are the first cross-sectional findings that try to evaluate, with specific laboratory tasks instead of rating-based measures, a developmental trend of inhibitory and delay of gratification performance in individuals with DS, considering both their mental age (MA) but especially their chronological age (CA). Furthermore, our research design and our findings are based on a theoretical model tested both in TD children and in people with DS [27,28].

Concerning the first aim of our research, our sample with DS showed overall greater difficulties in tasks tapping on diverse inhibitory components and delay of gratification considering accuracy score, while a mixed pattern emerged for response time (RT). Particularly, the sample with DS, compared to TD children with the same MA, performed worse on the Circle Drawing task (CDT), which requires the ability to suppress and control an impulsive motor response. It is salient to stress that people with DS had difficulties in aspects of fine and gross motor functioning and motor planning [4,76] and that fine motor control is highly associated with higher-order cognitive control in this population [77]. Therefore, given the crucial role of such abilities for a variety of everyday life activities, our results agree with the previous studies, which indicate the importance of assessing and monitoring the specific trajectory of motor response inhibition in individuals with DS [4]. Moreover, evidence indicates that there is a specific relationship between EFs and motor skills also in people with DS [48] and that fine motor integration could have important implications also on academic achievement because the brain uses a shared specific neural pathway between the cerebellum and prefrontal cortex [78].

Concerning the Preschool Matching Familiar Figure task (PMFFT) and the Grass/snow task, results indicated that individuals with DS are less accurate than TD children [28,79]. While the group with DS showed longer RT on the PMFFT, no differences emerged on the Grass/snow task. This result may suggest that people with DS, as early preschoolers, are not completely able to control RTs even in order to be more accurate and that given that RT may also be subject to greater noise for people with DS, it should be cautiously considered as a reliable index of executive control in this population [28,65,80]. Furthermore, it is important to consider the fact that both tasks require greater WM skills (compared, for example, to the CDT), and we speculate that the impairments of individuals with DS with both verbal and spatial-simultaneous stimuli could be related to their worse performance on these tasks [7,53,81]. For this reason, it would be necessary, in future research, to monitor WM ability and its possible effects on these tasks given the role of WM as a predictor of different inhibitory dimensions [30].

Different results emerged, also, for the three blocks of the Go/no-go task, with significant differences reported for the second and the third block and no differences on the first block on the no-go responses. While Traverso et al. [28] and Costanzo et al. [53] found no difference on this task between the group with DS and the TD control group, we considered all the three blocks separately to investigate people with DS performance on this task more in-depth. We think that the last two blocks could be more challenging for people with DS partly because they had to remember to inhibit their impulsive responses and to simultaneously also remember different rules with higher WM and shifting demand [82]. For the Go/no-go task, RTs are in line with the accuracy score reporting no differences on the first block between the two groups.

Considering interference suppression skills, no significant differences emerged between the two groups on accuracy score [28]. Contrary to our results on the Flanker task, Hauser-Cram et al. [83] and Merrill and O’dekirk [84] identified worse performances on the Fish Flanker task accuracy score and longer RT for the group with DS when they were faced with incongruent trials. However, it should be noted that both studies did not match the TD group with the sample with DS for a measure of MA and that the second study also used verbal stimuli. For the Hearts and Flowers task, worse performances were identified in both TD and DS groups, but it should be considered that this task measures complex inhibitory abilities and may require higher levels of WM skills than the Flanker task because subjects had to actively maintain the goal and the rules of the task in WM. However, despite the fact that in children of 5 to 6 years of age, response inhibition is distinguished from interference suppression [28], we speculate that the Hearts and Flowers task could be challenging not only for individuals with DS but also for TD children in this chronological age range. Nevertheless, this mixed pattern of results is in line with studies that highlighted the high variability on cognitive tasks in the atypical population (e.g., [85]).

Finally, people with DS showed worse performance compared to TD children also on the delay of gratification tasks (i.e., the Wrap Delay and the Marshmallow tasks). Our results are in line with previous research that evidenced difficulties in delaying gratification in individuals with DS [54,57]. Literature suggested that difficulties on the delay of gratification in individuals with DS could be due to: (a) specific language difficulties of people with DS (for a review see [86]) that also impaired the self-talk skills [55]; (b) poorer response inhibition and cool EFs such as WM [87]; (c) an overactivation of the hot system as a result of the faster development of the hot system that is not correctly modulated by the immature cool system ([88], pp. 83–105); (d) poor ability to integrate motivation with more abstract concepts and representations [89] such as the concept of time and the ability of time management that is strongly related to low levels of impulse control and poor resistance to distractions [90].

As mentioned above, the second aim of our study was to better understand inhibitory and delay of gratification abilities in individuals with DS with the same MA, in light also of their CA. For this reason, we considered the performances of two groups of the sample with DS: children and adolescents (DS1) and adults (DS2). Our results indicated that considering accuracy scores, the group DS1 performed worse—compared to the group DS2—on overall response inhibition and delay of gratification tasks, while no differences emerged between the two groups on the more complex interference suppression dimension. These results suggest that simpler inhibitory components (i.e., response inhibition and delay of gratification) may improve with age, whereas the more complex component (i.e., interference suppression) still remains also impaired in adulthood in people with DS. Previous studies that assess inhibition with a cross-sectional perspective suggested that these skills may ameliorate with age with a progressive decline after mid-30s [42,43]. It should be considered that both studies used only rating-based measures instead of laboratory tasks and that inhibition was not investigated considering specific sub-components. Our results are also in line with the Compensation Age Theory [91], which postulates that CA could influence and determine the cognitive abilities of individuals with intellectual disabilities beyond their MA. In fact, we think that levels of maturation and cumulative life experiences may explain better performance on inhibitory measures and the acquirement of suitable strategies to cope with new challenges in daily life that require the ability to know how to inhibit, to manage interference, and to wait to receive a reward or to carry out the desired action (see also [92]). Finally, contrary to Borella et al. [51], our results suggested the importance of considering different inhibitory dimensions. In fact, while Borella et al. [51] demonstrated a general inhibition impairment in people with DS, our results indicated specific impairments on the more complex inhibitory dimension that would remain more compromised even in adults with DS.

### 4.1. Limitations and Future Directions

To the best of our knowledge, our study is the first to assess, with a wide battery of tasks, response inhibition, interference suppression, and delay of gratification in people with DS, at first matching for MA the sample with DS with a TD control group, and secondly cross-sectionally analyzing differences on inhibitory sub-components between younger and older people with DS. Nevertheless, our research also has some limitations, such as the fact that our sample was too small in size to allow us to further divide it into a huge number of clusters with different CA ranges. Moreover, our attempt to cross-sectionally explore the developmental trajectories of inhibitory abilities in people with DS should warrant longitudinal replications. As a third point, it should be mentioned that response inhibition and interference suppression account differently on WM performances in TD children of 5 years of age, but only the interference suppression component served as a significant predictor for all WM tasks administrated. Given the paucity of studies focusing on interference suppression dimension in individuals with DS, future studies should consider the possibility of assessing this dimension using different tasks that capture more complex components taking into account both CA and MA implications and the specific cognitive profile of this population.

Even acknowledging these limitations, we believe that our study: (1) proposed a broad battery of tasks basing on a specific theoretical model [27,28] and tapping on response inhibition, interference suppression, and delay of gratification abilities; (2) suggested a developmental perspective of different inhibition abilities analyzing how age/experience could influence inhibitory skills.

Given that inhibition, together with WM, is an important predictor of academic achievement, adaptive behavior, daily life autonomy, and occupational perspectives [8,39], future studies should also jointly consider these dimensions in order to ensure overall better quality of life. Finally, we think that our results, which are in line with previous studies that indicated a growing path of EFs (e.g., [42,43]), raise important issues about how to set training programs or developmental paths for this population. While studies on TD children demonstrated that both cool and hot EFs could be improved [93,94], to the best of our knowledge, no training programs have yet been created and implemented to improve both components at the same time in individuals with DS. In fact, given the different developmental trajectories of diverse inhibitory sub-components and delay of gratification, we believe that it is important to propose training programs, both as regards the type of materials presented and the activities proposed, which take into account not only the MA of the individuals with DS but also the developmental CA trends of the specific function trained.

## 5. Conclusions

Comparing the group of individuals with DS with a TD control group matched for a measure of MA, our results indicate that the group with DS showed impaired performance in response inhibition, interference suppression, and delay of gratification. However, when younger individuals with DS were compared with older persons with DS, the older group with DS showed greater performance on both response inhibition and delay of gratification, while the interference suppression still remained impaired.

## Figures and Tables

**Table 1:** Descriptive statistics of measures and results of the comparisons among groups (MANOVA) for the two groups (DS vs. TD) for inhibitory tasks and delay of gratification tasks.

	Groups	N	Mean	SD	Min-Max	*F*	*p*	Effect Size (Range)
CDT	DS	51	0.34	0.27	0.61–0.80	49.12	0.0001 ***	0.29 (0.18–0.39)
	TD	71	20.58	3.80	12–25			
PMFFT Errors	DS	51	12.94	7.55	0–30	5.39	0.022 *	0.04 (0.003–0.11)
	TD	71	10.25	5.23	2–24			
PMFFT Time	DS	51	170.46	136.88	30.94–636.71	5.12	0.025 *	0.04 (0.003–0.11)
	TD	71	128.72	62.62	47.84–399.04			
Grass/snow Accuracy	DS	51	10.47	5.58	0–16	26.50	0.0001 ***	0.18 (0.09–0.28)
	TD	71	14.10	1.73	8–16			
Grass/snow Time	DS	51	36.37	10.39	22.25–77.02	2.03	0.157	0.02 (0–0.07)
	TD	71	34.30	5.45	25.55–49.86			
Go/no-go 1 Accuracy	DS	51	5.14	1.63	0–6	2.34	0.128	0.02 (0–0.08)
	TD	71	5.51	1.04	0–6			
Go/no-go 1 Time	DS	51	835.47	1839.17	0–7443	0.29	0.588	0.002 (0–0.04)
	TD	71	658.62	1729.35	0–7976			
Go/no-go 2 Accuracy	DS	51	3.00	1.78	0–6	47.75	0.0001 ***	0.28 (.017–0.38)
	TD	71	4.94	1.33	0–6			
Go/no-go 2 Time	DS	51	3348.71	2231.44	0–8314	33.09	0.0001 ***	0.22 (0.11–0.31)
	TD	71	1139.31	1986.75	0–12186			
Go/no-go 3 Accuracy	DS	51	4.25	2.29	0–8	77.61	0.0001 ***	0.39 (0.28–0.48)
	TD	71	7.04	1.16	2–8			
Go/no-go 3 Time	DS	51	3686.24	2602.06	0–11264	4.18	0.043 *	0.03 (0.0005–0.10)
	TD	71	4548.96	2054.10	0–11571			
Fish Flanker Accuracy	DS	51	13.35	3.77	3–16	3.70	0.057	0.03 (0–0.09)
	TD	71	14.58	3.23	1–16			
Fish Flanker Time	DS	51	35902.61	16548.86	11307–86529	3.93	0.05 *	0.07 (0.00002–0.10)
	TD	71	30530.56	12674.24	15683–72647			
Hearts and Flowers Accuracy	DS	51	5.35	3.41	0–10	1.15	0.285	0.01 (0–0.06)
	TD	71	4.65	3.70	0–10			
Hearts and Flowers Time	DS	51	20384.86	15145.43	5927.00–75480	3.02	0.085	0.03 (0–0.09)
	TD	71	16766.06	7518.67	4995.00–37842			
Wrap delay Latency time	DS	51	32.07	23.95	1.14–60	15.09	0.0001 ***	0.11 (0.04–0.20)
	TD	71	46.91	18.22	10.00–60			
Marshmallow Waiting time	DS	51	206.19	117.14	11.8–300	23.86	0.0001 ***	0.17 (0.07–0.26)
	TD	71	283.91	55.51	60.2–300			

Legend: CDT (Circle Drawing Task); PMFFT (Preschool Matching Familiar Figure Task); DS (Down Syndrome); TD (Typical Development). Note: * *p* < 0.05; *** *p* < 0.0001. Time is reported in seconds for the Preschool Matching Familiar Figure task time (PMFFT time) and in milliseconds for the PMFFT, Fish Flanker, and Hearts and Flowers task.

**Table 2:** Descriptive statistics of measures and results of the comparisons among groups (MANCOVA) for the two groups (DS1 vs. DS2) for CPM, inhibitory tasks, and delay of gratification tasks.

	Groups	N	Mean	SD	Min-Max	*F*	*p*	Effect Size
CPM	DS1	21	19.57	5.9	12–35	0.19	0.66	0.01
	DS2	30	18.90	5.03	11–28			
CDT	DS1	21	14.95	8	1–28	2.77	0.07	0.10
	DS2	30	11.53	7.02	0–30			
PMFFT Errors	DS1	21	126.54	118.82	30.94–580.21	9.02	0.0001 ***	0.27
	DS2	30	201.21	142.10	37.40–636.71			
PMFFT Time	DS1	21	9.10	5.16	0–16	2.26	0.12	0.09
	DS2	30	11.43	5.75	0–16			
Grass/snow Accuracy	DS1	21	39.03	11.34	26.22–77.02	5.00	0.01 *	0.17
	DS2	30	34.51	9.42	25.25–64.19			
Grass/snow Time	DS1	21	4.19	2.16	0–6	1.92	0.16	0.07
	DS2	30	5.80	0.48	4–6			
Go/no-go 1 Accuracy	DS1	21	1866.43	2538.44	0–7443	9.77	0.0001 ***	0.29
	DS2	30	113.80	282.18	0–1104			
Go/no-go 1 Time	DS1	21	2.33	1.98	0–6	7.98	0.001 **	0.25
	DS2	30	3.47	1.48	0–6			
Go/no-go 2 Accuracy	DS1	21	3143.27	2115.81	0–8314	6.71	0.003 **	0.22
	DS2	30	1139.31	1986.75	0–12186			
Go/no-go 2 Time	DS1	21	3.38	2.25	0–8	1.39	0.26	0.05
	DS2	30	4.87	2.15	0–8			
Go/no-go 3 Accuracy	DS1	21	4346.67	2613.77	0–11264	12.19	0.0001 ***	0.34
	DS2	30	3223.93	2534.79	0–11189			
Go/no-go 3 Time	DS1	21	12.86	3.60	3–16	6.29	0.004 **	0.21
	DS2	30	13.70	3.91	3–16			
Fish Flanker Accuracy	DS1	21	37921.22	16288.31	16520–67841	1.21	0.31	0.05
	DS2	30	34604.93	16879.96	11307–86529			
Fish Flanker Time	DS1	21	5.52	3.46	0–10	0.31	0.74	0.01
	DS2	30	5.23	3.42	0–10			
Hearts and Flowers Accuracy	DS1	21	16618.43	9356.85	5927–48740	1.06	0.35	0.04
	DS2	30	23021.37	17822.64	8058–75480			
Hearts and Flowers Time	DS1	21	25.64	22.15	3.08–60	1.39	0.26	0.05
	DS2	30	36.57	24.50	1.14–60			
Wrap delay Latency time	DS1	21	152.35	122.12	16.90–300	5.50	0.007 **	0.19
	DS2	30	243.87	99.02	11.80–300			
Marshmallow Waiting time	DS1	21	152.35	122.12	16.9–300	4.53	0.02 *	0.16
	DS2	30	243.87	99.02	11.80–300			

Legend: CPM (Coloured Progressive Matrices), CDT (Circle Drawing Task); PMFFT (Preschool Matching Familiar Figure Task); DS1 (children and adolescents with Down Syndrome); DS2 (adults with Down Syndrome). * *p* < 0.05; ** *p* < 0.001; *** *p* < 0.0001. Time is reported in seconds for the Preschool Matching Familiar Figure task time and in milliseconds for the PMFFT, Fish Flanker, and Hearts and Flowers tasks. CPM score as a measure of nonverbal reasoning score was used as a covariate.

## Data Availability

Not applicable.
